# Establishment of an immortalized yak granulosa cell line: *in vitro* tool for understanding the molecular processes of ovarian follicle development

**DOI:** 10.3389/fvets.2024.1362502

**Published:** 2024-04-24

**Authors:** Xiao Wen, Jia Zhou, Shuangming Yue, Jun Zhang, Yuanyuan Chen, Zhisheng Wang, Lizhi Wang, Quanhui Peng, Bai Xue

**Affiliations:** ^1^Institute of Animal Nutrition, Sichuan Agricultural University, Chengdu, China; ^2^Department of Bioengineering, Sichuan Water Conservancy Vocation College, Chengdu, China; ^3^Qinghai Academy of Animal Science and Veterinary Medicine, Qinghai University, Xining, China

**Keywords:** yak, granulosa cell, immortalization, cell line, follicular development

## Abstract

The yak, a unique species of cattle found exclusively on the western plateau of China, is a valuable source of livelihood for local residents. However, their low fecundity restricts the expansion of yak farming, whereas regional factors limit studies on yak breeding. Granulosa cells (GCs), which provide essential steroid hormones and growth factors for oocytes, have been the focus of many studies on the mechanisms of follicular growth and atresia. This study aimed to establish an immortalized cell line model that could serve as a tool for future studies on the mechanisms of ovarian follicle development in yaks. First, we isolated primary yak granulosa cells (yGCs) and evaluated their replicative senescence after continuous *in vitro* subculturing. Subsequently, an immortalized culture method for primary yGC was explored, and a new cell line model was established to study the mechanism of follicular development *in vitro*. We used a mammalian gene expression lentivirus vector to transfer the simian virus 40 large T antigen (SV40T) into primary yGC to obtain an immortalized cell line. The immortalized yGCs were morphologically identical to the primary yGCs, and cell proliferation and growth were normal within a limited number of generations. Follicle-stimulating hormone receptor (FSHR), a specific marker for GCs, was positively expressed in immortalized yGCs. Furthermore, the immortalized yGCs retained the ability of GCs to synthesize estradiol and progesterone and expressed genes related to steroid synthesis. The establishment of immortalized yGC opens up a myriad of possibilities for advancing our understanding of yak reproductive biology and improving yak breeding strategies.

## Introduction

Yaks (*Bos grunniens*) are primarily distributed in the plateau regions of western China at altitudes of 2,500–6,000 m, where they provide meat, dairy products, transportation, and fuel (dung) to the local and surrounding populations (1, 2). Yaks play an indispensable role in the lives of people living in the mountainous regions. However, the low reproductive efficiency of yaks, which typically breed once every 2 years or twice every 3 years, as a result of seasonal breeding, delayed puberty, and low estrus frequency, restricts the development of the yak industry (1). The limitations of yak grazing habits and living environments have resulted in fewer studies on yak reproduction than those conducted on dairy cows and beef cattle. The majority of the available research has primarily focused on animal experiments, with only a few investigations delving into the molecular mechanisms *in vitro*.

Successful ovulation is a prerequisite for a successful pregnancy. Gonadal steroids and other factors regulate follicular development (3). During follicular development, the vast majority of follicles degenerate with atresia, accompanied by granulosa cell apoptosis (4, 5). GCs synthesize and secrete steroid hormones, growth factors, and cytokines that regulate the microenvironment within the follicle (6). The proliferation, apoptosis, and steroid synthesis capacity of GCs determine the fate of the follicles (continued growth or atresia) (7). Thus, GCs have been used as a valuable approach for studying ovarian follicle biology *in vitro* (8). In mammals, almost all normal somatic cells experience delayed proliferation, growth arrest, and loss of differentiation after limited division *in vitro* (9, 10). This phenomenon, called replicative senescence, is the ultimate outcome of primary cells dividing *in vitro* (11, 12). Primary culture is the sole option to obtain yak GCs (yGCs); however, the high cost of collecting yak ovaries further limits the exploration of molecular mechanisms at the cellular level.

Various methods have been developed to obtain persistently proliferating cells for use in *in vitro* model systems, including spontaneous immortalization, oncogenic transformation, radiation-induced tumorigenesis, and chemical mutagenesis. These methods have been used to establish immortalized cells such as “Hela Cells” (13). The introgression of lentivirus genes, such as simian virus 40, human papillomavirus, Epstein–Barr virus, or other viruses, into primary cells to induce tumor characteristics is a common method for establishing immortalized cell lines (14, 15). The simian virus SV40 is a rhesus polyomavirus that was first identified in 1960. Its genome encodes two proteins: a large T antigen and a small T antigen (16). The simian virus 40 large T antigen (SV40T) has been widely used for primary cell immortalization by regulating the p53-mediated cell cycle (17). The senescence of normal primary cells *in vitro* is partly due to the gradual shortening of cell telomeres (18). Transferring exogenous human telomerase reverse transcriptase into primary cells, theoretically activates cellular telomerase to elongate telomeres, maintain chromosomal stability, allow cells to pass the M1 and M2 stages smoothly, and close to divide and proliferate indefinitely, resulting in prolonged cell culture time or immortalization *in vitro* (14, 19). Recently, the transfer of SV40T into primary cells has successfully established immortalized cell lines that retain the morphological and functional characteristics of primary cells (20, 21). Although stable granulosa cell lines have been established from the primary GCs of humans (22), bovines (23), pigs (24), and rats (25), no studies have been conducted on yaks. Thus, this study evaluated the changes in replicative senescence properties of primary yGCs during long-term *in vitro* expansion. Furthermore, this study established an immortalized yGCs for the first time, providing material for *in vitro* experiments and serving as a model for further studies on the molecular mechanisms of follicular development in yaks.

## Materials and methods

### Ethics statement

This study was conducted in accordance with guidelines established by the Animal Care and Use Committee of China. The experimental procedures were approved by the Animal Ethics and Welfare Committee of Sichuan Agricultural University (#SCAUAC2020-84).

### Isolation, cultivation, and passage of primary yak GCs

The isolation and culture of primary yGCs were based on a previous report, with minor modifications (26). Briefly, the ovaries of yak heifers were collected from Qinghai Yutai Livestock Products Co., Ltd. (Xining, China) and transported to the laboratory within 3 h at a constant temperature (approximately 37°C) in phosphate-buffered saline (PBS; Solarbio, Beijing, China) containing 5% streptomycin and penicillin (Gibco, Shanghai, China). Follicular fluid was aspirated from 2 to 8 mm small follicles using a 2.5 mL sterile syringe (Honghu Taining Medical Devices Co., Ltd., Jingzhou, China), placed in a 15-mL sterile centrifuge tube (NEST Biotechnology, Wuxi, China), and centrifuged at 300 × *g* for 3 min to obtain the precipitate. The precipitate was resuspended in PBS (containing 2% streptomycin and penicillin), preheated at 37°C, filtered through BeyoGold™ 40 μm cell strainers (Beyotime, Shanghai, China), and centrifuged at 300 × *g* for 3 min to remove impurities. The final cell precipitate was resuspended in Dulbecco’s Modified Eagle’s Medium and Ham’s F12 Nutrient Mixture (DMEM/F12, Gibco, Carlsbad, CA, USA) supplemented with 10% fetal bovine serum (Gibco, Shanghai, China) and 1% streptomycin and penicillin. The resuspended yGC was transferred to a T25 flask (NEST) at a density of 1 × 10^5^ cells/mL and cultured in an incubator (Thermo Fisher Scientific, Waltham, MA, USA) at 37°C and 5% CO_2_. The medium was replaced every 48 h to prevent nutrient depletion. When yGCs reached approximately 85% confluence, they were passaged at a 1:2 ratio.

### Establishment of an immortalized yak GC line

The HBLV-ZsGreen-Puro vector was purchased from VectorBuilder (Guangzhou, China), and the HBLV-SV40T-3*flag-ZsGreen-Puro lentiviral expression vector was constructed and packaged. The method for establishing immortalized yGC was based on a previous report with minor modifications (27). Briefly, when the yGC reached 50% confluency, the culture medium was changed to DMEM/F12 with multiple infection numbers (MOIs) of 80 and 5 mg/L of polybrene (Sigma, St. Louis, MO, United States). After overnight infection with the lentivirus, yGCs were cultured in a fresh medium for 4 days. Subsequently, the yGCs were cultured in a medium containing 2 mg/L of puromycin for 4 days to screen for surviving cell lines, which were designated as immortalized yGCs. The medium was changed every 48 h, unless otherwise specified.

### Cell viability assay

Primary (passage 1) and immortalized (passage 12) yGCs were seeded at a density of 2 × 10^3^ cells/well in 96-well plates (NEST) and cultured overnight. Cell viability was determined using the Cell Counting Kit 8 (CCK-8, APExBio, Houston, TX, United States) on days 0, 1, 2, 3, 4, 5, and 6, with six replicates per time point. The culture medium in each well was replaced with a mixture (100 μL) containing 90% serum-free DMEM/F12 and 10% CCK-8 solution and incubated at 37°C for 2 h. The absorbance of each well at 450 nm and 650 nm was measured using a SpectraMax M2 microplate reader (Molecular Devices, Sunnyvale, CA, United States). Three wells without cells were used as blank controls.

### Senescence assessment

Primary yGCs (passages 1, 5, and 7) and immortalized (passages 7, 15, and 23) yGCs were incubated in 6-well plates (NEST) for 48 h. The medium was discarded, and the cells were washed with PBS and fixed in 4% paraformaldehyde (Beyotime) for 15 min at room temperature. Next, the cells were stained with a senescence β-galactosidase (SA-β-Gal) staining kit (Beyotime) at 37°C for 12 h, following the manufacturer’s instructions. The presence of senescent cells triggered by the activity of a specific senescence-associated β-galactosidase was identified under a light microscope by observing the resulting dark blue product.

### Immunofluorescence identification

Immunofluorescence of ovarian tissue, yGCs, and negative controls (without additional primary antibodies) was performed according to the previous reports, with minor modifications (27, 28). Briefly, fresh yak ovaries were fixed in 4% paraformaldehyde for 24 h and embedded in wax, sliced, and dewaxed before antigen retrieval. Primary (passage 1) and immortalized (passage 10) yGCs were incubated in 6-well plates for 24 h and then fixed with 4% paraformaldehyde for 15 min. The ovarian tissue and cells were sealed with 5% goat serum (Solarbio, Beijing, China) and incubated with an anti-follicle-stimulating hormone receptor (FSHR) antibody (Bioss, Beijing, China). Immortalized yGCs were incubated with an anti-SV40T-antigen antibody (Abcam, Cambridge, MA, United States) for 12 h at 4°C. The target protein was subsequently labeled with fluorescein isothiocyanate- (FITC-) or sulfo-cyanine 3 (CY3)-conjugated goat anti-rabbit IgG secondary antibodies (Bioss) for 2 h. The cell nuclei were stained with 4′,6-diamidino-2-phenylindole (DAPI; Solarbio, Beijing, China) for 2 min and then observed under a fluorescence microscope (Nikon, Japan).

### Hormone concentration assay

Primary (passage 1) and immortalized (passage 10) yGCs were incubated in 6-well plates until 80% confluency was reached. Thereafter, the medium was replaced with fresh culture medium, and the cells were cultured for 24 h before collection. The concentrations of estradiol and progesterone in the culture medium were determined using commercially available ELISA kits (Meimian, Jiangsu, China), following the manufacturer’s instructions.

### RNA extraction and qPCR analysis

Total RNA from primary (passage 1) and immortalized (passage 10) yGCs was extracted and purified using a total RNA extraction kit (Accurate; Changsha, China). The concentration and purity of the extracted RNA were determined using a NanoDrop 2000 spectrophotometer (Thermo Fisher Scientific). Subsequently, the qualified RNA was reverse-transcribed to cDNA using a reverse transcription kit (ExonScript RT SuperMix with dsDNase; Exongen, China). The synthesized cDNA was diluted to 1:4 with enzyme-free water (Sangong, Shanghai, China). Quantitative polymerase chain reaction (qPCR) was performed using the 2 × Fast SYBR Green qPCR Master Mix Kit (Servicebio, Wuhan, China) in the Applied Biosystems^™^ QuantStudio^™^ 5 Real-Time PCR System (Thermo Fisher Scientific) according to the manufacturer’s instructions. The primer sequences used in the qPCR analysis are listed in Table 1, with β-actin serving as the housekeeping gene. The relative mRNA abundance of target genes was determined using the comparative cycle threshold (2^−ΔΔCt^) method.

**Table 1:** Information on the primers used in qPCR.

Gene	Sequence (5′–3′)	GenBank accession number	Product length (bp)
*CYP51A1*	F: CGGGATAGTGATGCTGGACT	XM_005908819.1	126
	R: ACCAGACTAAGCGTGAAGGC		
*ARG2*	F: ACCCCTCACCACTTCATCTG	XM_005905728.1	159
	R: GGGTCCACGTCTCTTAGTCC		
*CYP19A1*	F: GGTGTCCGAAGTTGTGCCTA	XM_005900467.1	146
	R: ACCTGCAGTGGGAAATGAGG		
*CYP11A1*	F: CCGAGGCCCAGAAGTTCATT	XM_005905795.2	137
	R: AATTGTGTCCCATGCGGCTA		
*HSD17B1*	F: CCGTCCCAGAGCTTCAAAGT	XM_005899199.1	136
	R: AATCTGCATCCCTCACGTCC		
*HSD17B8*	F: AACTTGGACGACACGGGATC	XM_005891389.1	166
	R: GCCAAGAATGCGACGACATC		
*ESR1*	F: CAAGAACGTGGTGCCTCTCT	XM_014476936.1	190
	R: GCTGGGGAAATTCTCTGCCT		
*ESR2*	F: TCCTATGTAGAGAGCCGCCA	XM_014478950.1	180
	R: AACTGATGGCTGGCAATGGA		
*β-actin*	R: TCACACATCCTTCAGCACCT	XM_005887322.2	102
	F: TGGCGCTTGACTCAGGATTT		

### Statistical analysis

Statistical significance was determined using an independent sample t-test with SPSS v23.0 (SPSS Inc., Chicago, IL, United States), and the findings are presented as the mean and standard error. A *p-*value of <0.05 was considered statistically significant.

## Results

### Isolation and culture of the primary yak GCs

**Figure 1:**
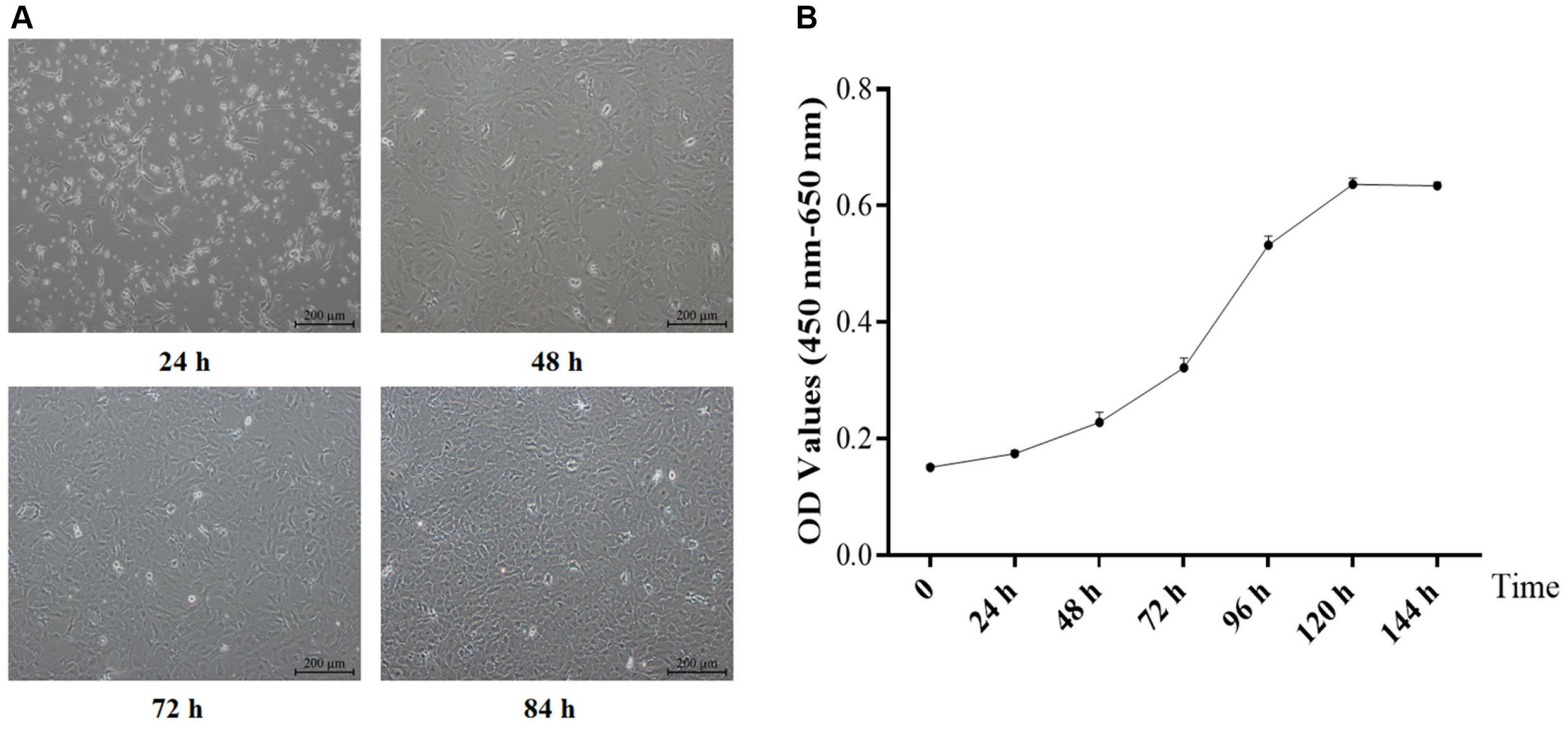
Morphology (**A**, Scale bars = 200 μm) and growth curve **(B)** of the primary GCs (*n* = 6). OD, optical density values.

The formation of typical “paving stones” monolayer cells (Figure 1A) characterized the transition of primary yGCs into a stationary density state. The growth curve of primary yGC (Figure 1B) exhibited an S-shaped trajectory culminating in a plateau at 120 h.

### Immunofluorescence identification of the primary yak GCs

**Figure 2:**
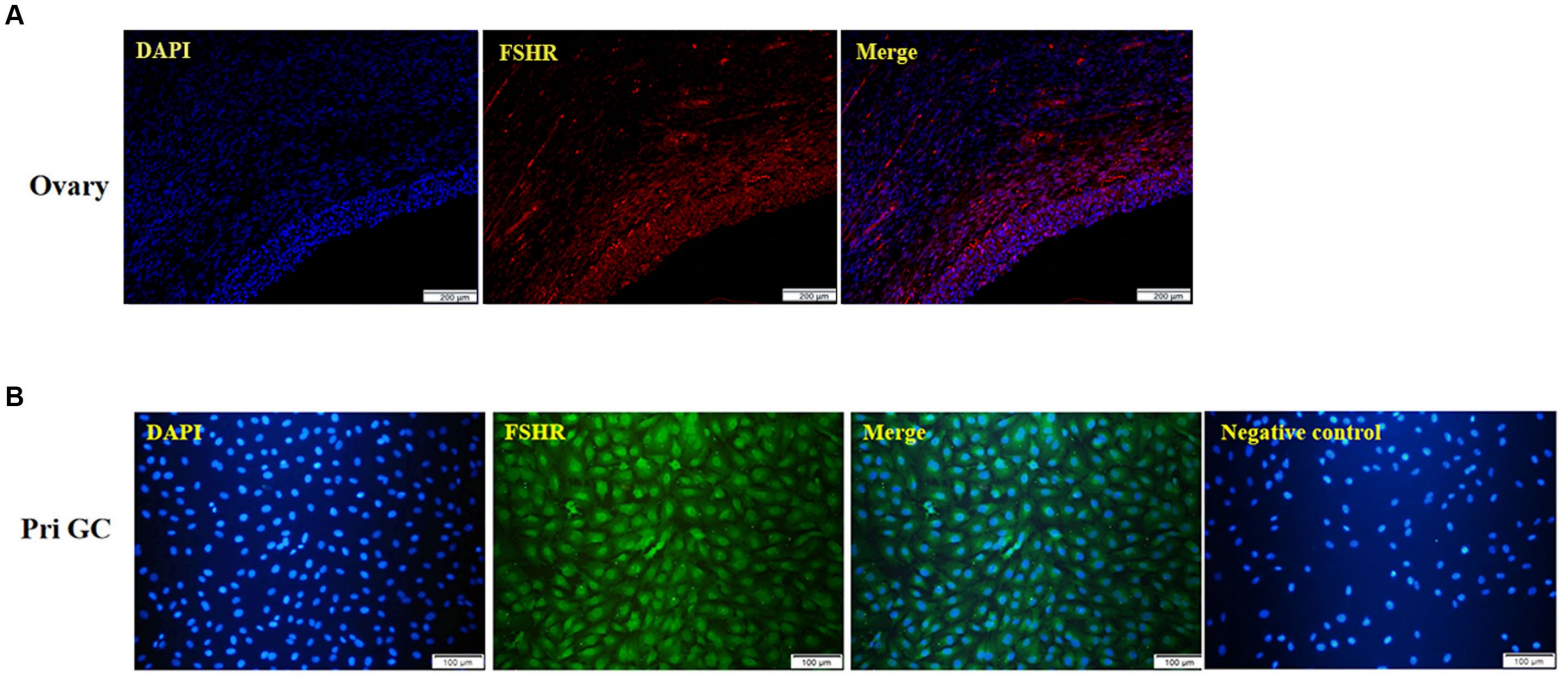
Immunofluorescence of the yak ovary (**A**, scale bars = 200 μm) and primary GCs (**B**, scale bars = 100 μm). Pri GC, primary granulosa cells.

Immunofluorescence analysis of yak ovaries revealed that the FSHR protein was specifically expressed in ovarian follicles (Figure 2A). Furthermore, cell immunofluorescence revealed that the isolated cultured cells were yak GCs, with over 85% of the cells in the field of view expressing the FSHR protein (Figure 2B).

### Senescence assessment of primary yak GCs

**Figure 3:**
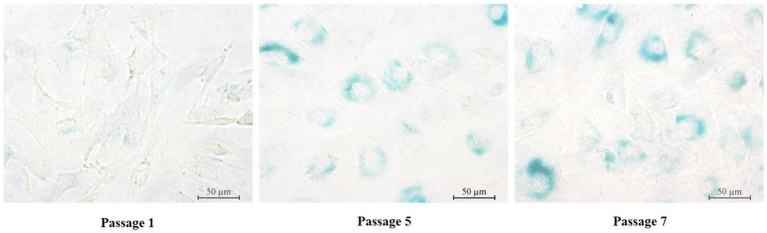
SA-β-Gal staining in primary GCs of different generations. Pri GCs, primary granulosa cells. Scale bars = 50 μm.

SA-β-Gal staining has been widely used to evaluate cellular senescence. The results of this study demonstrated that primary yGCs from the first passage stained negatively for SA-β-Gal (Figure 3A). However, the fifth-generation primary yGCs displayed positive staining for SA-β-Gal, as indicated by the appearance of blue-colored areas within the cells (Figure 3B). Furthermore, the seventh generation primary yGCs showed distinctly positive SA-β-Gal staining, as evidenced by the midnight blue color areas within almost all cells (Figure 3C). These findings revealed that primary yGCs exhibited replicational senescence in the late passage.

### Characteristics of immortalized yak GCs

**Figure 4:**
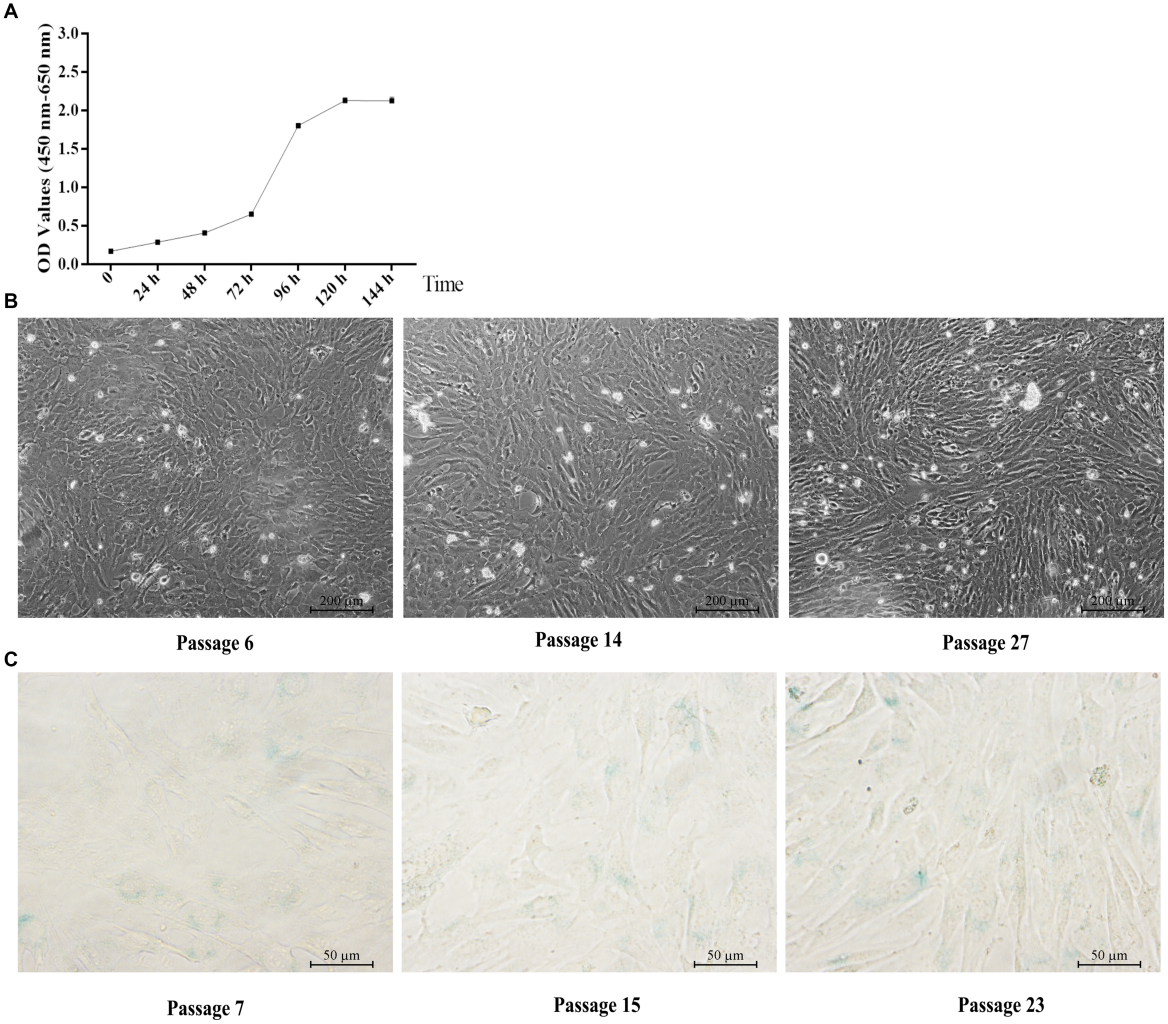
Characteristic of immortalized yGCs. **(A)** Growth curve of immortalized yGCs. **(B)** Morphology of immortalized yGCs at passages 6, 14, and 27 (*n* = 6). OD, optical density values. Scale bars = 200 μm. **(C)** SA-β-Gal staining in immortalized yGCs at passages 7, 15, and 23. Scale bars = 50 μm.

Similar to the primary yGCs, the growth curve of the immortalized yGCs followed a characteristic S-shape, as shown in Figure 4A. The immortalized yGCs retained the morphological features of the primary yGCs across different generations (Figure 4B). Furthermore, negative staining for SA-β-Gal indicated that immortalized yGCs lost the replicative senescence characteristics of the primary yGCs within a limited number of generations (Figure 4C).

### Identification of immortalized yak GCs

**Figure 5:**
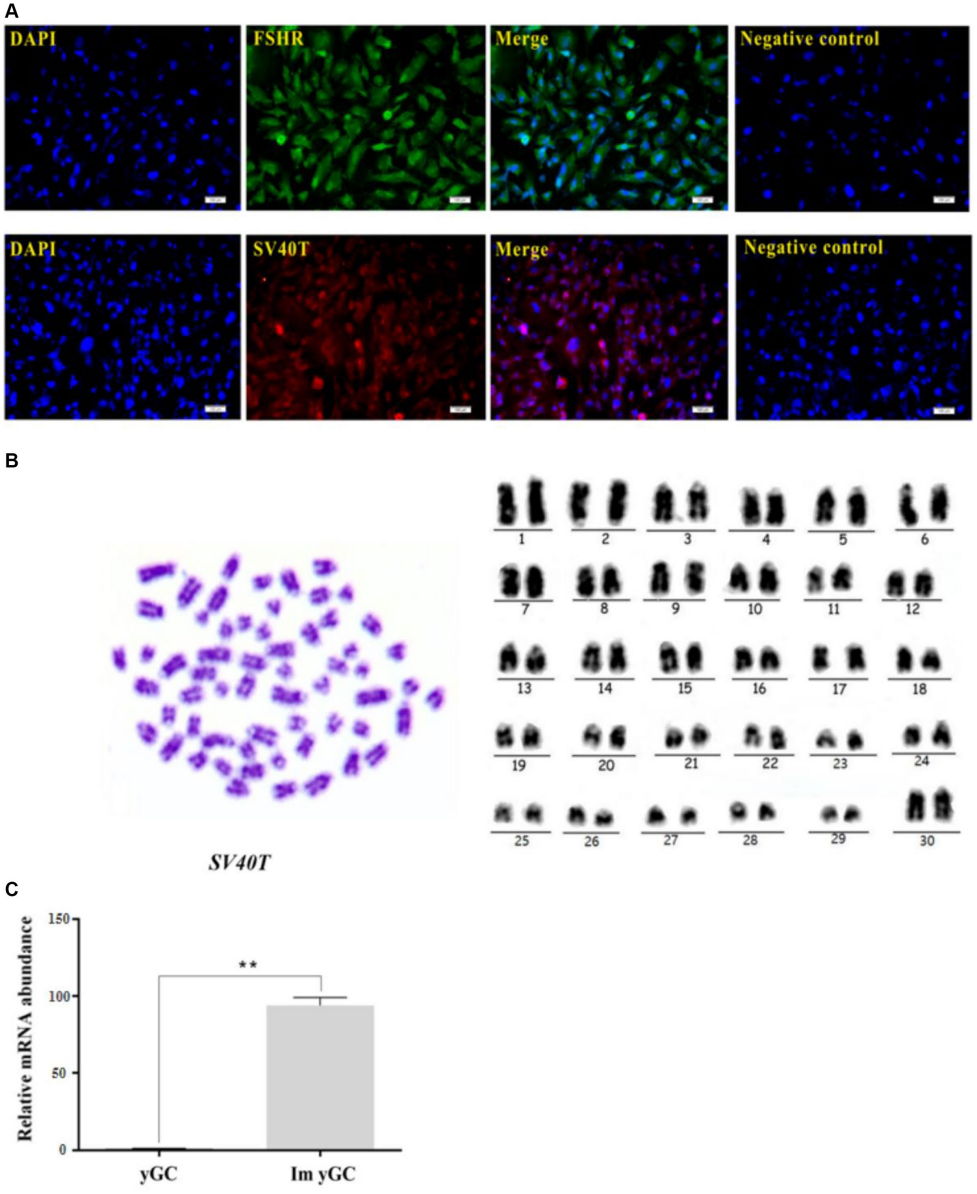
Identification of immortalized yGCs. **(A)** Immunofluorescence of FSHR in immortalized yGCs, scale bars = 100 μm. **(B)** Immunofluorescence of SV40T in immortalized yGCs, SV40T, simian virus 40 large T antigen. Scale bars = 100 μm. Cytogenetic analysis of immortalized yGCs. **(C)** The mRNA abundance of SV40T in the primary yGCs and immortalized yGCs; Pri yGCs; primary yak granulosa cells, Im yGC, immortalized yak granulosa cell line. **p < 0.01.

The immortalized yGCs, similar to the primary yGCs, showed strong immunoreactivity to the FSHR protein (Figure 5A). Chromosomal karyotype analysis was used to confirm the origin of the cell line based on cytogenetic characterization. Chromosomes were analyzed, compared, ranked, and numbered based on chromosome length, centromere position, ratio of long and short arms, and presence or absence of followers. The results revealed that the immortalized yGCs had a diploid karyotype consistent with the chromosomal characteristics of the yak species (2n = 60), including 29 pairs of autosomes and one pair of sex chromosomes (Figure 5B). The immortalized yGCs were infected with lentivirus expressing SV40 large T antigen, which was produced by co-transfecting the lentiviral vector (HBLV-SV40T-3*flag-ZsGreen-Puro) with packaging vectors (Figure 5B). The immortalized yGCs had a significantly higher abundance of SV40T mRNA than the primary yGCs (*p* < 0.05) (Figure 5C).

### Functional analysis of immortalized yak GCs

**Figure 6:**
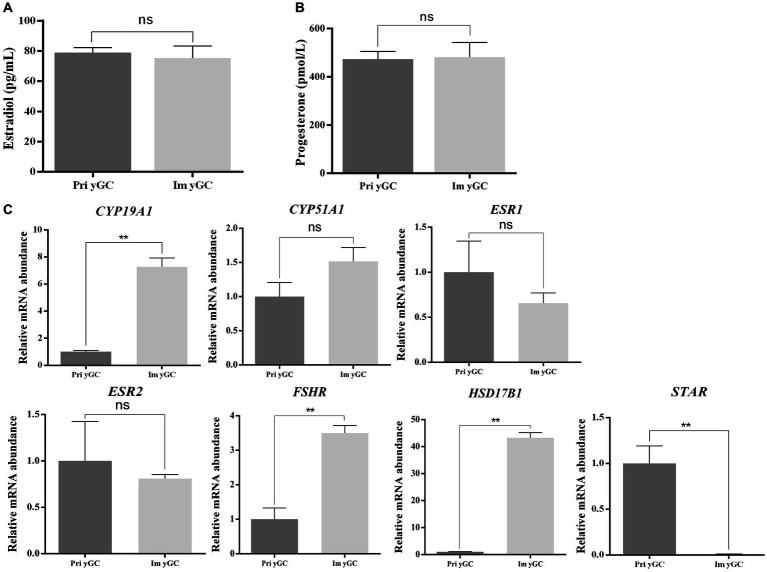
Functional identification of immortalized yGCs. **(A)** Differences in estradiol secretion between primary GC and immortalized yGCs. **(B)** Differences in progesterone secretion between primary GCs and immortalized yGCs. **(C)** Differences in the expression of genes related to steroid hormone synthesis between primary GCs and immortalized yGCs. Pri yGCs, primary yak granulosa cells; Im yGC, immortalized yak granulosa cell line (*n* = 3), ** *p* < 0.01.

There was no difference in the concentrations of estradiol and progesterone in the culture media of primary and immortalized yGCs (Figures 6A,B). However, the mRNA expression of *CYP19A1*, *FSHR,* and *HSD17B1* was significantly higher in immortalized yGCs than in primary yGCs (*p* < 0.05). In contrast, *STAR* mRNA expression was significantly higher in primary yGCs than in immortalized yGCs (*p* < 0.05) (Figure 6C).

## Discussion

Immortalized cells are defined as cells that have been modified to bypass the normal restrictions on the cell cycle, allowing them to proliferate indefinitely. This process typically involves the introduction of viral or oncogene-mediated alterations to the cell genome, which can result in uncontrolled cell proliferation and tumor formation. In fact, diploid cells have a low possibility of immortalization *in vitro* under natural conditions (29, 30). However, the use of slow-transcription viral vectors has enabled the successful transfer of immortalized genes into primary cells, which can avoid the issues of proliferation crisis and senescence *in vitro* while maintaining their physiological and biochemical characteristics (31, 32). In the present study, the SV40T gene was introduced into primary yGCs to establish an immortalized cell line.

First, the primary yGCs were successfully isolated and cultured. Previous studies have confirmed the identity of yGCs through their morphological structure (paving stones) and immunofluorescence (specific FSHR expression) (33, 34). In the previous *in vitro* studies on yGCs, second-generation cells were used for experimental purposes (33–35). In the previous bovine GC culturing procedure (36), the rate of cell proliferation declined at passage 14 and cell growth plateaued at passage 7 (particularly for cryopreserved and re-thawed cells). SA-β-Gal staining revealed that the primary yGCs were partially aged at the fifth passage and almost all of them were aged at the seventh passage, indicating that the degree of cell senescence gradually increased with the number of generations. In bovine GCs, replicative senescence began at the 14th passage with a strong positive SA-β-Gal staining and arrested the cell cycle in the G0/G1 phase (36). However, further research is needed to understand the relationship between replicational senescence and the GC passage number. Replicative senescence occurs earlier in yGCs than is reported in bovine GCs, highlighting the significance of yGC immortalization.

Ovarian GCs are the primary functional cells of the ovary, and their proliferation and differentiation directly affect ovarian functions such as follicle growth initiation, development, ovulation, corpus luteum formation, and steroid hormone secretion (37). In adult animals, one of the most significant signs of follicular development is the rapid growth and proliferation of GCs. Follicular atresia is mostly caused by GC apoptosis, particularly in the late stages of follicular development (38). Additionally, GCs are responsible for synthesizing certain steroid hormones that maintain the milieu for oocyte growth and maturation, the most significant of which is estrogen (39). In this study, immortalized yGCs were successfully constructed and characterized. Previously, immortalized yGCs were found to have hormone secretion functions similar to those of the primary yGCs. Although genes related to steroid hormone synthesis were expressed in both primary and immortalized yGCs, the expression levels of several genes varied. Consistent with previous studies, there are differences in the expression levels of certain genes and proteins between immortalized cells constructed using SV40T and primary cells (40, 41). Overall, the immortalized yGCs constructed in this study retained the morphological and functional characteristics of primary yGCs, making them suitable for *in vitro* studies of follicle development.

## Conclusion

This study presents a framework for the efficient generation of immortalized yGCs to overcome the constraints of replicative senescence in primary yGCs. Immortalized yGCs possess functional characteristics similar to those of primary cells and the ability to proliferate indefinitely. The application of these immortalized yGCs will significantly contribute to *in vitro* studies on the regulation of yak follicle development.

## Data availability statement

The original contributions presented in the study are included in the article/supplementary material, further inquiries can be directed to the corresponding author.

## Ethics statement

This study was conducted in accordance with guidelines established by the Animal Care and Use Committee of China. The experimental procedures were approved by the Animal Ethics and Welfare Committee of Sichuan Agricultural University (#SCAUAC2020-84).

## Author contributions

XW: Writing – original draft, Validation, Methodology. JiZ: Writing – original draft, Methodology, Investigation, Conceptualization. SY: Writing – review & editing, Methodology. JuZ: Writing – review & editing, Software, Methodology. YC: Writing – original draft, Validation, Methodology. ZW: Writing – review & editing, Software, Conceptualization. LW: Writing – review & editing, Methodology. QP: Writing – review & editing, Methodology. BX: Writing – review & editing, Supervision, Funding acquisition, Conceptualization.
